# Exploring causal associations between dietary intake and liver diseases: A bidirectional Mendelian randomization study

**DOI:** 10.1097/MD.0000000000040095

**Published:** 2024-11-08

**Authors:** Hong Wang, Zhangjun Yun, Liling Li, Hui Wang, Haotian Zeng, Yun Ran

**Affiliations:** a Dongzhimen Hospital, Beijing University of Chinese Medicine, Beijing, China; b Beijing University of Chinese Medicine Third Affiliated Hospital, Beijing, China; c Shenzhen Hospital, Beijing University of Chinese Medicine, Shenzhen, China.

**Keywords:** cirrhosis, Genome-wide association studies, Mendelian randomization, poultry, salad

## Abstract

Previous evidence suggests that dietary intake can affect liver diseases; However, the causal relationship between dietary intake and liver diseases remains unclear. To investigate this, we conducted a bidirectional Mendelian randomization (MR) analysis to comprehensively assess the potential causal relationship between dietary intake and liver diseases. Two-sample bidirectional MR was performed based on genome-wide association studies summary data from the UK Biobank and FinnGen database. The primary analysis method for evaluating causal relationships was inverse-variance weighted. Supplementary analyses included MR-Egger and weighted median methods. Subsequently, sensitivity analyses were performed using Cochran *Q* test, MR-Egger intercept test, MR-PRESSO, RadialMR, and leave-one-out analysis to assess heterogeneity and horizontal pleiotropy. MR evidence indicated that genetically predicted poultry intake (adjusted odds ratio [OR] = 0.04, 95% confidence interval [CI] = 0.00–0.43, *P* = .007) and salad/raw vegetable intake (adjusted OR = 0.18, 95% CI = 0.04–0.83, *P* = .028) were directly associated with a reduced risk of cirrhosis. Conversely, there is no causal association between dietary intake and nonalcoholic fatty liver disease, alcoholic liver disease, or hepatocellular carcinoma. This study provides evidence supporting the impact of dietary intake on liver disease. Increased intake of poultry and salad/raw vegetables is associated with a reduced risk of cirrhosis. These findings can inform preventive and therapeutic strategies for cirrhosis.

## 1. Introduction

Liver diseases encompass a series of digestive system diseases that primarily affect the liver, such as alcoholic liver disease, cirrhosis, liver cancer, and nonalcoholic fatty liver disease (NAFLD). These conditions are often attributed to a combination of factors such as infections, immune system abnormalities, and genetic factors.^[1]^ Liver diseases constitute a significant cause of global mortality, contributing to approximately 2 million deaths annually.^[2]^ Despite various medical interventions and some level of efficacy achieved in managing different liver diseases, they remain a pressing public health concern, imposing substantial burdens on both population health and economies. Therefore, it is necessary to explore and understand its main risk factors in order to carry out targeted interventions for the prevention of liver diseases.

Numerous studies have indicated that dietary habits may play a crucial role in the occurrence and development of liver diseases. For example, a cohort study in Finland found that consuming alcohol excessively at least once a month (≥5 drinks per occasion) was associated with an increased risk of liver diseases. Additionally, binge drinking showed a positive correlation with the risk of liver diseases, and individuals who binged every week have the highest risk.^[3]^ There is also evidence indicating a direct relationship between alcohol intake (exceeding the threshold of 30 g per day) and the likelihood of developing alcoholic liver disease.^[4]^ A high-sugar diet is identified as a high-risk environmental factor influencing the onset and progression of NAFLD, leading to reduced hepatic steatosis but increased fibrosis.^[5]^ Moreover, research has shown the role of diet in the development of liver cancer. For example, the intake of tea, coffee, vegetables, and cereal fiber may contribute to lowering the risk of liver cancer, whereas excessive alcohol consumption (more than approximately 3 drinks per day), high dairy intake (3 servings per day or more), and processed meat consumption could increase the risk of liver cancer.^[6]^ However, the presence of various confounding factors, such as environmental changes and lifestyle differences, may introduce bias in clinical studies estimating the relationship between dietary intake and liver diseases. Hence, further elucidation is required to establish the causal relationship between the two.

Mendelian randomization (MR) has become a commonly employed research method in recent years, examining causal relationships between exposure and outcome by using genetic variations as instrumental variables.^[7]^ According to Mendel laws, alleles of single nucleotide polymorphisms (SNPs) segregate randomly and independently of environmental factors,^[8]^ which to a large extent mitigates the bias introduced by confounding factors in observational studies. Two-sample MR analysis was employed in this study to explore the potential causal associations between dietary intake and liver diseases. This opened up new prospects for the prevention and treatment of liver diseases in clinical practice.

## 2. Materials and methods

### 2.1. Study design

**Figure 1.**
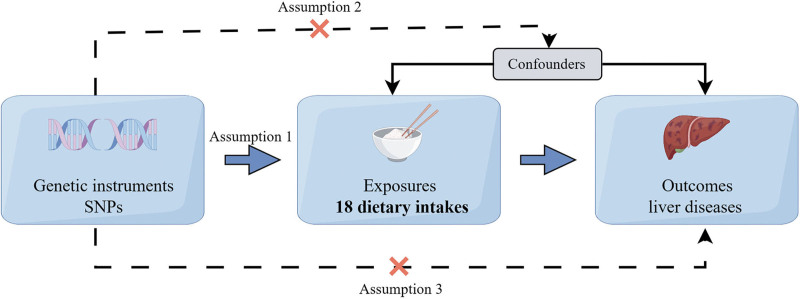
Mendelian randomization study design flowchart. The 3 core assumptions are as follows: (1) Assumption 1: the SNPs should be robustly associated with dietary intake; (2) Assumption 2: the SNPs should be independent of potential confounders; (3) Assumption 3: the SNPs should affect liver diseases exclusively through dietary intake. Solid arrows represent allowed relationships between variables, dashed lines and red crosses indicate relationships where genetic instruments are not allowed as valid instrumentals. By Figdraw (www.figdraw.com). SNPs = single nucleotide polymorphisms.

To assess the causal relationship between dietary intake and liver diseases, SNPs were selected as instrumental variables (IVs) for MR analysis. The SNPs used for the analysis must satisfy the 3 essential assumptions of IVs: (1) the IV is significantly associated with the exposure risk factor; (2) the IV is independent of potential confounding factors; (3) the IV affects the outcome exclusively through the exposure risk factor.^[9]^ The data used in this study were obtained from publicly available databases that have been ethically approved for the original research. Therefore, no additional ethical approval or informed consent was required for this study. The overall design of the MR study is shown in Figure 1.

### 2.2. Data source

**Figure 2.**
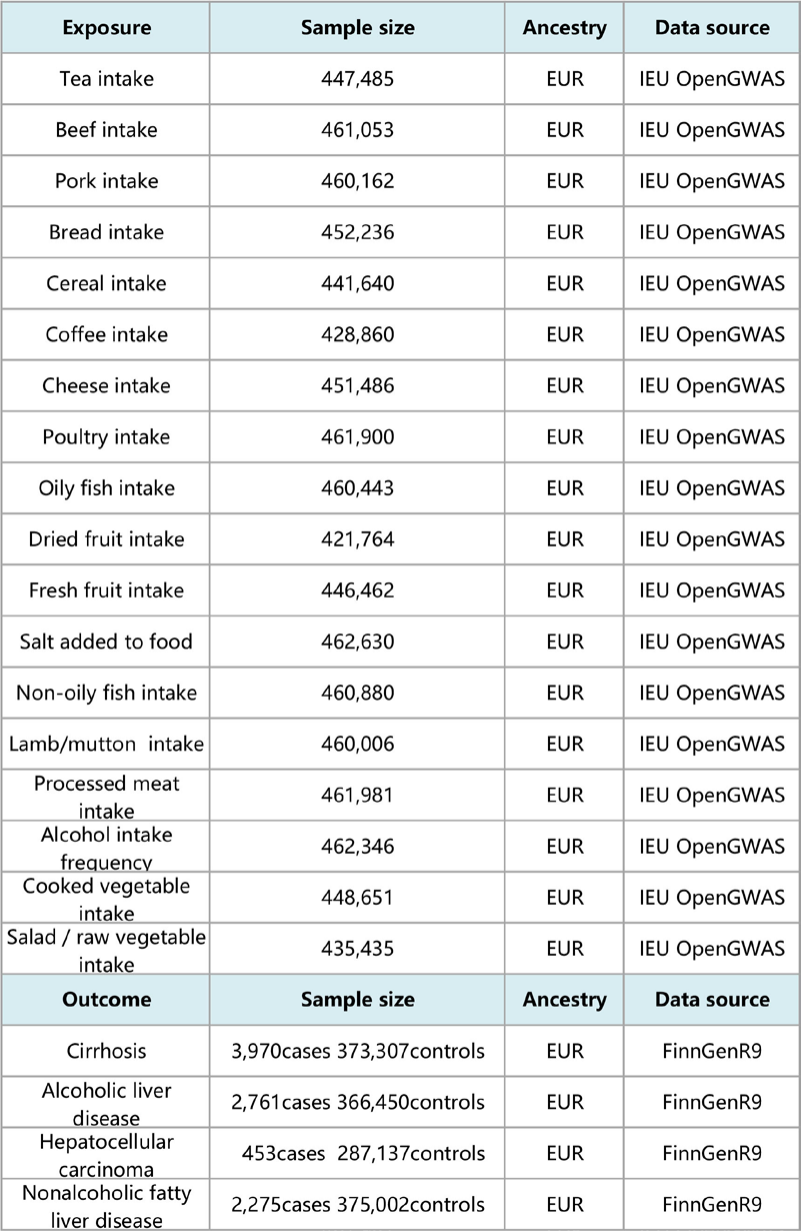
Detailed information on all GWAS datasets. EUR = European, GWAS = Genome-wide association studies.

Genome-wide association studies (GWAS) summary statistics for dietary intake were obtained from the UK Biobank, a large-scale prospective cohort that includes extensive phenotypic and genotypic details from approximately 500,000 participants across 22 assessment centers in the UK.^[10]^ We selected 18 dietary intake phenotypes as exposure datasets, including alcohol intake frequency, beef intake, bread intake, cereal intake, cheese intake, coffee intake, cooked vegetable intake, dried fruit intake, fresh fruit intake, lamb/mutton intake, non-oily fish intake, oily fish intake, pork intake, poultry intake, processed meat intake, salad/raw vegetable intake, salt added to food, and tea intake. To avoid sample overlap between exposure and outcome and the influence of racial differences, we obtained summary-level GWAS data on liver diseases in European populations from the FinnGen database (9th release).^[11]^ This dataset includes liver cirrhosis, alcoholic liver disease, hepatocellular carcinoma, and nonalcoholic fatty liver disease. Detailed information on all GWAS datasets used in this study is provided in Figure 2.

### 2.3. Instrumental variables selection

To meet the key assumptions of MR, we implemented a series of quality control procedures to select eligible instrumental variants. First, we extracted SNPs with *P* < 5 × 10^−8^, which are considered to be significantly associated with the exposure at the genome-wide level. Next, we used the European sample from the 1000 Genomes Project as the reference panel to perform linkage disequilibrium clumping, removing SNPs with *R*² < 0.001 within a 10,000 kb window to ensure the independence of the SNPs.^[12]^ Third, to eliminate bias from weak instruments, we calculated the *F*-statistic for each SNP to assess the strength of the association between the SNPs and the exposure. SNPs with *F* < 10 were defined as weak instruments and were removed.^[13]^ To exclude potential confounding factors, we investigated each SNP in the instrument variables using the GWAS Catalog database (https://www.ebi.ac.uk/gwas/), removing SNPs that were significantly associated with the outcomes (*P* < 5 × 10^−8^). Finally, we harmonized the SNPs for both exposure and outcome, removing palindromic SNPs and allelic inconsistent SNPs, ensuring the consistency of alleles between exposure and outcome.

### 2.4. Statistical analysis

We conducted a two-sample MR analysis to assess the causal relationship between dietary intake and liver diseases. Inverse-variance weighted (IVW) analysis was used as the primary analysis method. IVW assumes that all SNPs meet the 3 key MR assumptions and there is no pleiotropy.^[14]^ Under these conditions, IVW provides the most accurate estimation of causal effects. To obtain more robust results, we complemented the analysis with MR-Egger and the weighted median method. The MR-Egger method can provide a reliable causal effect estimation under the InSIDE assumption (Instrument strength independent of direct effect) and assess directional pleiotropy using the MR-Egger regression intercept test.^[15]^ The weighted median method can offer a robust causal effect estimation even if over 50% of the SNPs are invalid instruments.^[16]^

To further ensure the robustness of our research results, we performed several sensitivity analyses using various methods. Cochran *Q* test was used to evaluate heterogeneity, with a *P*-value >.05 indicating no heterogeneity.^[17]^ “RadialMR” was employed to identify and remove outlier pleiotropic SNPs.^[18]^ Additionally, leave-one-out analysis was conducted to determine whether the overall causal effect was driven by any single SNP.^[19]^ We also used MR-PRESSO to detect and correct horizontal pleiotropy and to identify and remove outliers.^[20]^ Subsequently, reverse MR was employed to validate the independent causal relationships identified in our significant outcomes.

All data processing and statistical analyses for MR were conducted using the R software (version 4.2.3) with the TwoSampleMR package (version 0.5.7), MR-PRESSO (version 1.0), and RadialMR package (version 1.1).^[18]^

## 3. Results

### 3.1. Characteristics of genetic variation associated with dietary intake

Table S1, Supplemental Digital Content, http://links.lww.com/MD/N746 provides detailed information on the specific SNPs used in the MR analysis. For all examined exposures, the *F*-statistic for SNPs exceeded the empirical threshold of 10, suggesting that the results of the MR analysis were less likely to be biased by weak instrumental variables.

### 3.2. Univariable MR analysis of dietary intake and liver diseases

**Figure 3.**
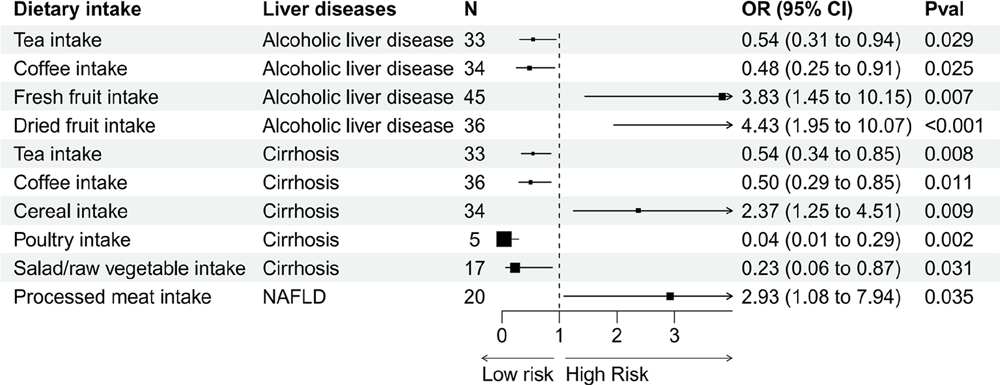
Assessment of the strength of potential causal associations between dietary intake and liver diseases. CI = confidence interval, N = number of SNPs, NAFLD = nonalcoholic fatty liver disease, OR = odds ratio, Pval = *P*-value, SNPs = single nucleotide polymorphisms.

The primary results of the univariable MR analysis were determined based on IVW analysis, supporting a causal relationship between dietary intake and liver diseases (Fig. 3).

This study found that genetically predicted tea intake (odds ratio [OR] = 0.54, 95% confidence interval [CI] = 0.31–0.94, *P* = .029) and coffee intake (OR = 0.48, 95% CI = 0.25–0.91, *P* = .025) were associated with a reduced risk of alcoholic liver disease. Conversely, increased consumption of fresh fruits (OR = 3.83, 95% CI = 1.45–10.15, *P* = .007) was nominally correlated with a higher risk of alcoholic liver disease, while dried fruit intake (OR = 4.43, 95% CI = 1.95–10.07, *P* < .001) was significantly associated with an elevated risk of alcoholic liver disease. We also observed that tea intake (OR = 0.54, 95% CI = 0.34–0.85, *P* = .008), coffee intake (OR = 0.50, 95% CI = 0.29–0.85, *P* = .011), poultry intake (OR = 0.04, 95% CI = 0.01–0.29, *P* = .002), and salad/raw vegetable intake (OR = 0.23, 95% CI = 0.06–0.87, *P* = .031) were associated with a reduced risk of cirrhosis. Conversely, cereal intake (OR = 2.37, 95% CI = 1.25–4.51, *P* = .009) exhibited an increased risk of cirrhosis to some extent. For NAFLD, the genetically predicted processed meat intake (OR = 2.93, 95% CI = 1.08–7.94, *P* = .035) often amplified disease risk. Furthermore, no significant associations were found between dietary intake and hepatocellular carcinoma. Complete MR analysis results are available in Table S2, Supplemental Digital Content, http://links.lww.com/MD/N746.

**Figure 4.**
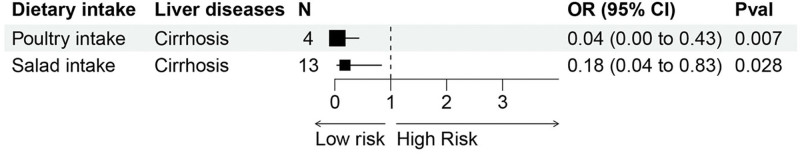
Assessment of the strength of potential causal associations between dietary intake and liver diseases (after correction for confounding factors). CI = confidence interval, N = number of SNPs, OR = odds ratio, Pval = *P*-value, SNPs = single nucleotide polymorphisms.

To mitigate the potential influence of confounding factors on the results of the study, we excluded SNPs closely related to confounders from all positive results and conducted univariable MR analysis again (Fig. 4). The results indicated that tea intake (adjusted OR = 0.98, 95% CI = 0.43–2.24, *P* = .960), coffee intake (adjusted OR = 1.12, 95% CI = 0.31–4.04, *P* = .867), dried fruit intake (adjusted OR = 2.88, 95% CI = 1.08–7.66, *P* = .034), and fresh fruit intake (adjusted OR = 2.88, 95% CI = 0.97–8.57, *P* = .058) were not statistically significantly associated with alcoholic liver disease. Similarly, there was no evidence supporting a causal relationship between tea intake (adjusted OR = 0.45, 95% CI = 0.23–0.89, *P* = .021), coffee intake (adjusted OR = 0.53, 95% CI = 0.20–1.47, *P* = .223), and cereal intake (adjusted OR = 1.96, 95% CI = 0.79–4.90, *P* = .149) with cirrhosis. Additionally, no causal relationship was found between processed meat intake (adjusted OR = 2.11, 95% CI = 0.73–6.10, *P* = .168) and NAFLD. This indicates that their associations may be influenced by confounding factors such as obesity, type 2 diabetes, and metabolic syndrome. However, poultry intake (adjusted OR = 0.04, 95% CI = 0.00–0.43, *P* = .007) and salad/raw vegetable intake (adjusted OR = 0.18, 95% CI = 0.04–0.83, *P* = .028) maintained a statistically significant relationship with cirrhosis even after adjustment for confounding factors. The specific information on confounding factors and the adjusted MR analysis results can be found in full in Tables S5 and S6, Supplemental Digital Content, http://links.lww.com/MD/N746.

Other analytical methods align directionally with the IVW method. Based on Cochran *Q* test and the MR-Egger regression intercept test (all *P*-values > .05, Table S3, Supplemental Digital Content, http://links.lww.com/MD/N746), there was no evidence of potential heterogeneity or pleiotropy bias in our results. Visual inspections through funnel plots and scatter plots did not reveal significant asymmetry or heterogeneity. Leave-one-out analysis further confirmed that the results were not significantly driven by any individual SNP. MR-PRESSO did not detect any potential outliers (all *P*-values > .05). Complete MR analysis results are available in Figures S1–S12, Supplemental Digital Content, http://links.lww.com/MD/N745.

### 3.3. Reverse MR analysis of dietary intake and liver diseases

In the reverse MR analysis, using IVW as the primary analysis, genetically predicted dietary intake showed no evidence of reverse causation with liver diseases. Other analytical methods provided consistent directional evidence, with no evidence of heterogeneity or pleiotropy. The results of all reverse MR analyses are shown in Table S4, Supplemental Digital Content, http://links.lww.com/MD/N746.

## 4. Discussion

This study employed bidirectional MR to systematically assess the causal relationship between 18 dietary intake factors and 5 liver diseases. The results indicate that a higher intake of dried fruits is associated with an increased risk of alcoholic liver disease, whereas the intake of salad/raw vegetables and poultry exhibits a protective effect against cirrhosis. Other MR analysis methods were consistent with the IVW method, suggesting the robustness of our results. Additionally, we addressed the issue of horizontal pleiotropy in causal estimates by making different assumptions, and the results indicate that horizontal pleiotropy is unlikely to significantly affect our findings. Furthermore, reverse MR analysis was conducted to explore the possibility of reverse causality between outcomes and exposures, but no substantial evidence was found to support the existence of reverse causality between liver diseases and dietary intake.

As living standards improve, significant changes have occurred in modern lifestyles and dietary patterns. Consumption of high-fat, high-sugar, and high-calorie foods has become mainstream, while consumption of healthy low-fat foods such as white meat, vegetables, and fruits has become less common. Poultry (such as chicken, duck, and goose) is categorized as white meat and typically contains lower levels of fat and cholesterol compared to red meat, while being rich in protein, which is essential for maintaining normal bodily functions. Research has found that white meat has less saturated fat and heme iron content and is rich in polyunsaturated fatty acid. n-3 polyunsaturated fatty acid can inhibit the synthesis of IL-1 and tumor necrosis factor, exerting anti-inflammatory activity, which helps protect liver cells from inflammation damage and prevent the occurrence of liver diseases.^[21]^ Another cohort study involving over 500,000 participants suggests that consuming white meat (poultry and fish) is associated with a reduced risk of liver disease-related mortality.^[22]^

Salads and raw vegetables typically have low-calorie content and are rich in dietary fiber and various vitamins. Dietary fiber helps control weight and reduce cholesterol levels, thereby alleviating the burden on the liver. Vitamins C and E, among others, exert antioxidant effects by neutralizing free radicals in the body, reducing oxidative stress-induced damage to the liver. Cohort studies have confirmed a close correlation between higher vegetable intake and lower liver fat content.^[23]^ Similarly, research has found that Siberian onions, as a type of vegetable, can reduce the release of inflammatory factors and inhibit STAT3 phosphorylation to alleviate liver fibrosis.^[24]^ The leaves of *Ficus lepicarpa* B, a traditional Malaysian medicine, when consumed as vegetables, can reduce the increase in serum ALT, AST, and liver MDA levels. They also inhibit the expression of pro-inflammatory cytokines TNF-α, IL-6, and prostaglandin E2, thereby treating cirrhosis in mice.^[25]^

This study has several key strengths: Firstly, MR has proven valuable in assessing causal inferences. Compared to traditional observational studies, MR research is less susceptible to reverse causation and potential confounding factors; and it is also less prone to random or systematic measurement errors. Moreover, it provides robust supportive evidence while requiring relatively less time and cost investment. Secondly, this research systematically examines the relationship between dietary intake and liver diseases, some of which were previously unreported in studies. Thirdly, we utilized liver diseases GWAS data from the FinnGen Consortium and dietary intake-related data from the UK Biobank, effectively averting population overlap between exposure and outcome, thereby minimizing the potential biases and type 1 error rates arising from sample overlap.^[26]^ All GWAS summary-level data used in this study are sourced from individuals of European ancestry, reducing population stratification bias and enhancing result stability. Lastly, the extensive sample size, and robust genetic tools (SNP *F*-statistic > 10), along with sensitivity and pleiotropy analyses, ensure the robustness of the study’s outcomes.

However, this study also presents several limitations. Firstly, due to the absence of GWAS summary data stratified by different age groups and genders, we were unable to conduct stratified analyses based on these factors. Secondly, all participants in this study belonged to individuals of European ancestry, potentially limiting the generalizability of our findings to non-European populations. Thirdly, MR analysis assumes that the effect of a genetic variant on an outcome is solely through exposure. However, there is a potential for other unmeasured factors to influence this relationship, which might result in horizontal pleiotropic associations that could be difficult to quantify and address. Lastly, despite our efforts to address the issue of weak instruments through various statistical tests, it is important to acknowledge that this issue may persist to some extent. As such, caution should be exercised when interpreting the results of our study, and further research is needed to confirm our findings and address potential sources of bias.

## 5. Conclusion

In summary, this MR study supports a causal relationship between dietary intake and liver diseases, indicating that higher consumption of poultry and salad/raw vegetables can reduce the risk of cirrhosis. Our findings provide valuable insights for dietary interventions in the prevention and management of cirrhosis. Future well-designed randomized controlled trials are necessary to validate these findings, and further research should be conducted to elucidate the underlying mechanisms.

## Acknowledgments

We extend our gratitude to the UK Biobank, FinnGen biobank, and the IEU OpenGWAS project for sharing the summary-level GWAS data and the collective efforts of researchers.

## Author contributions

**Conceptualization:** Hong Wang.

**Data curation:** Hong Wang, Zhangjun Yun, Hui Wang.

**Formal analysis:** Hong Wang, Zhangjun Yun, Liling Li.

**Funding acquisition:** Yun Ran.

**Methodology:** Hong Wang, Zhangjun Yun.

**Project administration:** Yun Ran.

**Resources:** Haotian Zeng.

**Software:** Hong Wang, Liling Li, Hui Wang.

**Supervision:** Yun Ran.

**Visualization:** Hong Wang, Hui Wang.

**Writing—original draft:** Hong Wang, Zhangjun Yun.

**Writing—review & editing:** Hong Wang, Zhangjun Yun, Liling Li, Haotian Zeng, Yun Ran.

## Supplementary Material


