# Supplementary material for: Exploring causal associations between dietary intake and liver diseases: A bidirectional Mendelian randomization study
Source: Medicine (Baltimore). 2024 Nov 8;103(45):e40095. doi: 10.1097/MD.0000000000040095 (PMC11557105; doi:10.1097/MD.0000000000040095)
Supplement: Supplementary file 2 [file medi-103-e40095-s002.docx]

**Supplementary Figure**


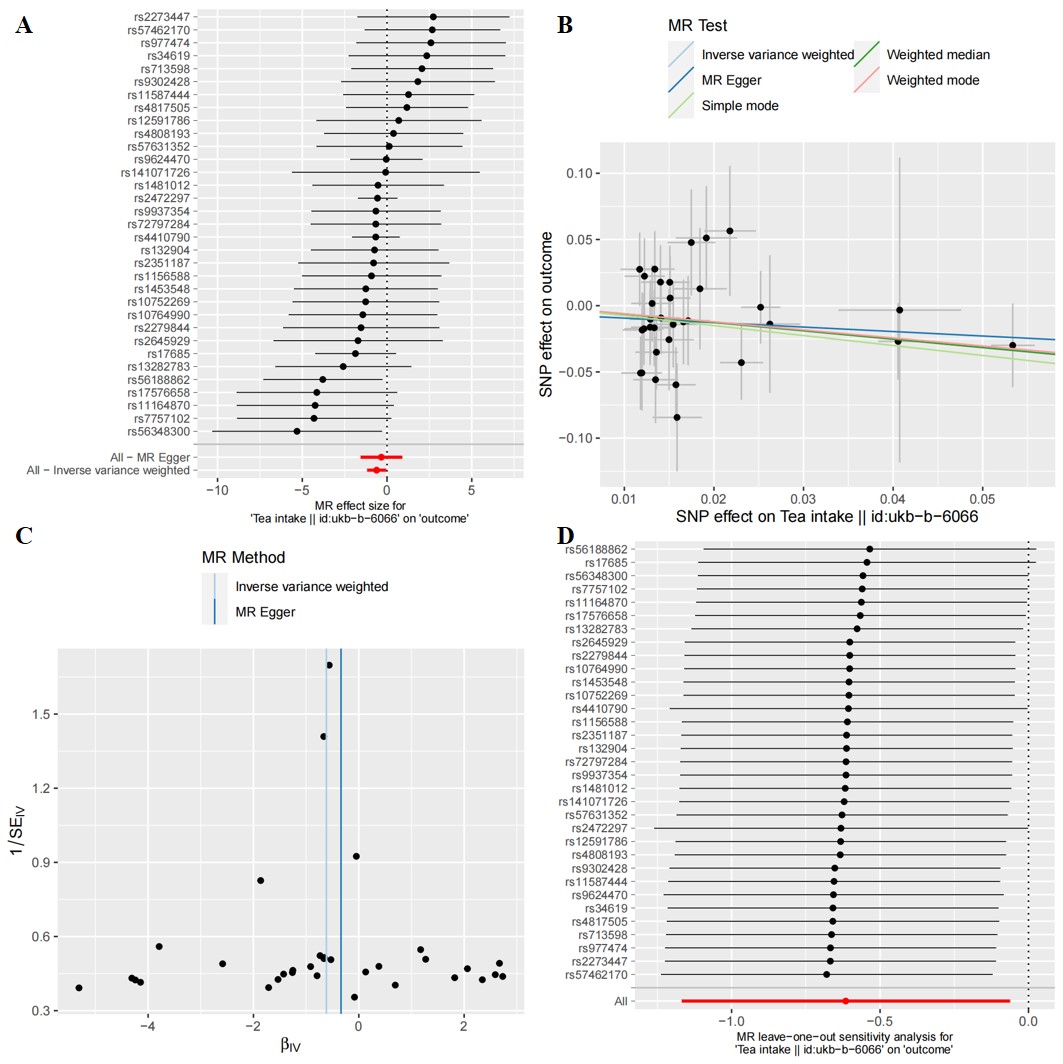


**Figure S1.** Forest plots (A), scatter plots (B), funnel plots (C), and leave-one-out plots (D) of genetically predicted tea intake on alcoholic liver disease.

**Supplementary Figure**


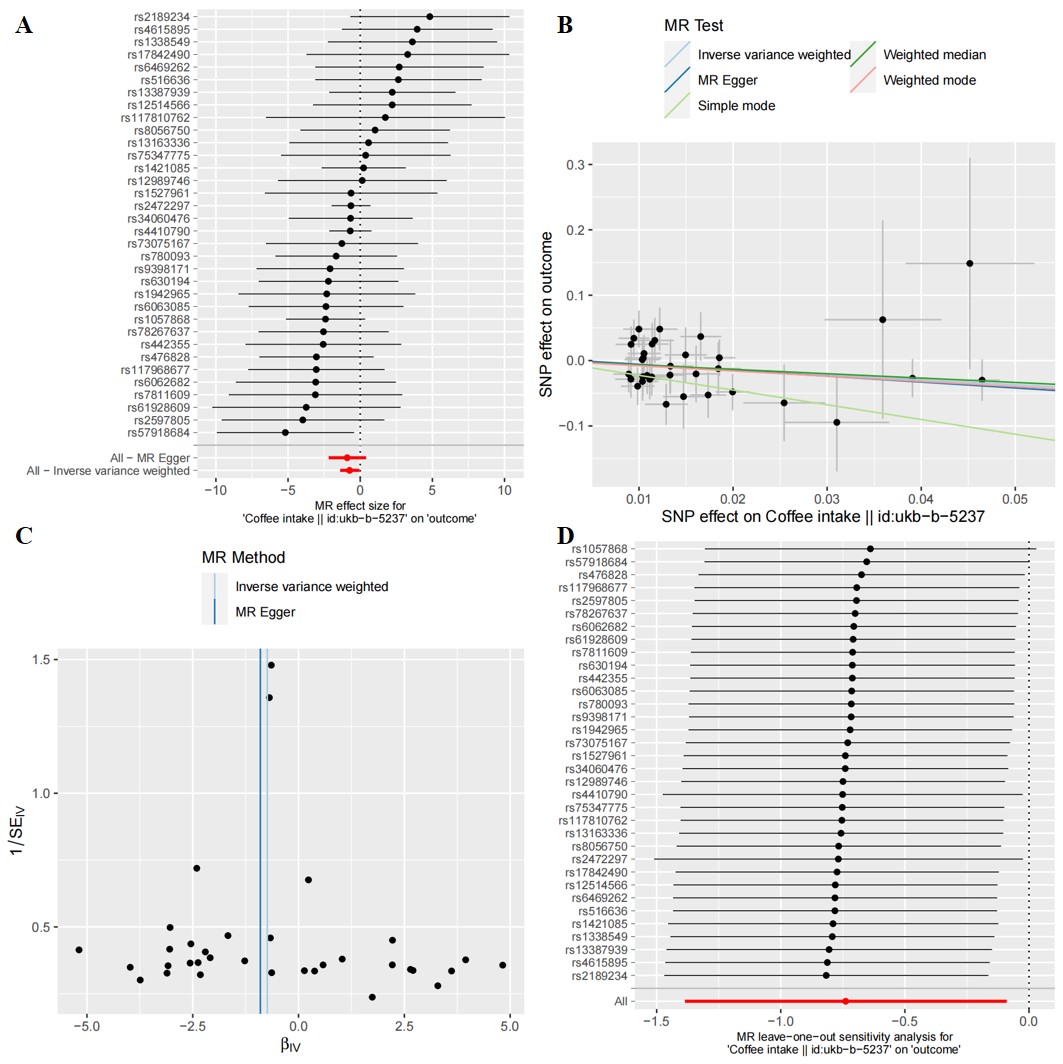


**Figure S2.** Forest plots (A), scatter plots (B), funnel plots (C), and leave-one-out plots (D) of genetically predicted coffee intake on alcoholic liver disease.

**Supplementary Figure**


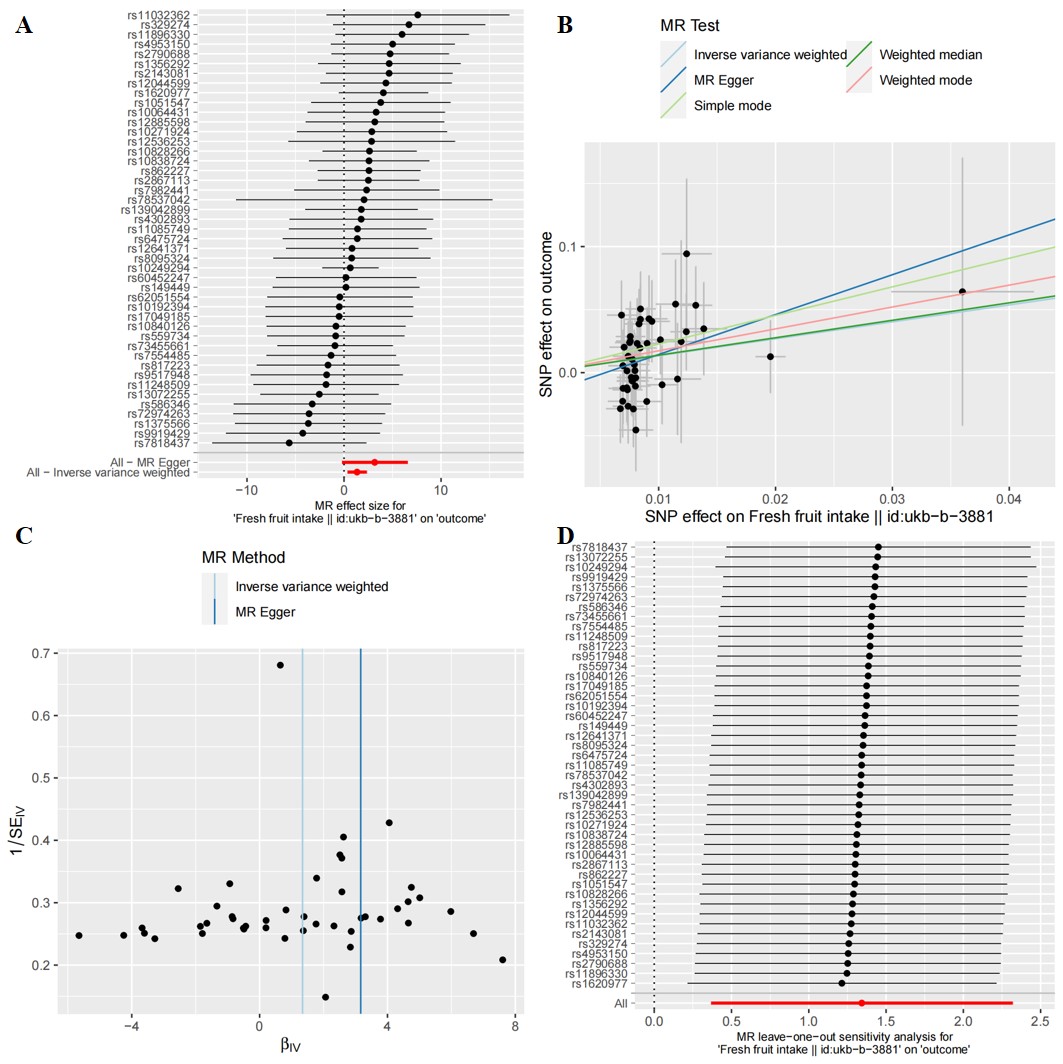


**Figure S3.** Forest plots (A), scatter plots (B), funnel plots (C), and leave-one-out plots (D) of genetically predicted fresh fruit intake on alcoholic liver disease.

**Supplementary Figure**


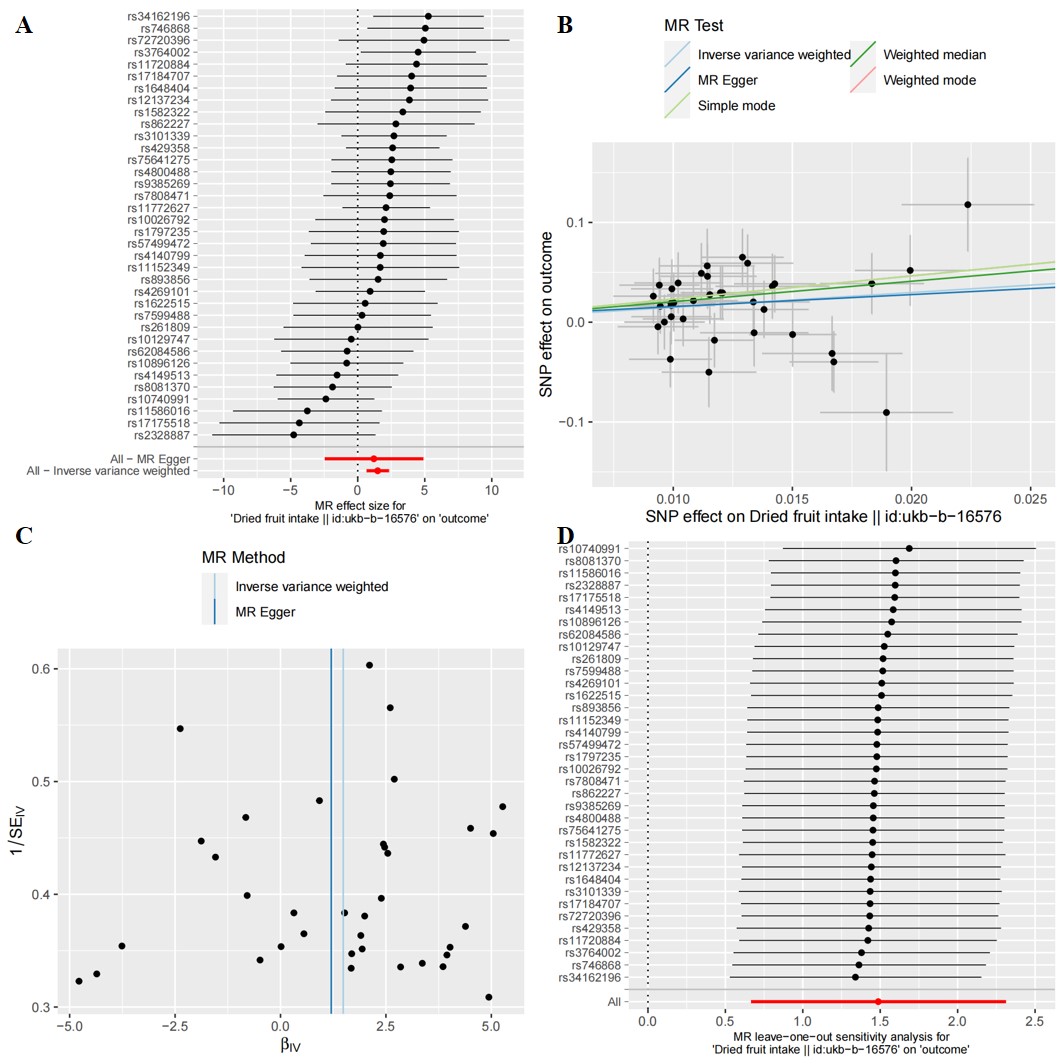


**Figure S4.** Forest plots (A), scatter plots (B), funnel plots (C), and leave-one-out plots (D) of genetically predicted dried fruit intake on alcoholic liver disease.

**Supplementary Figure**


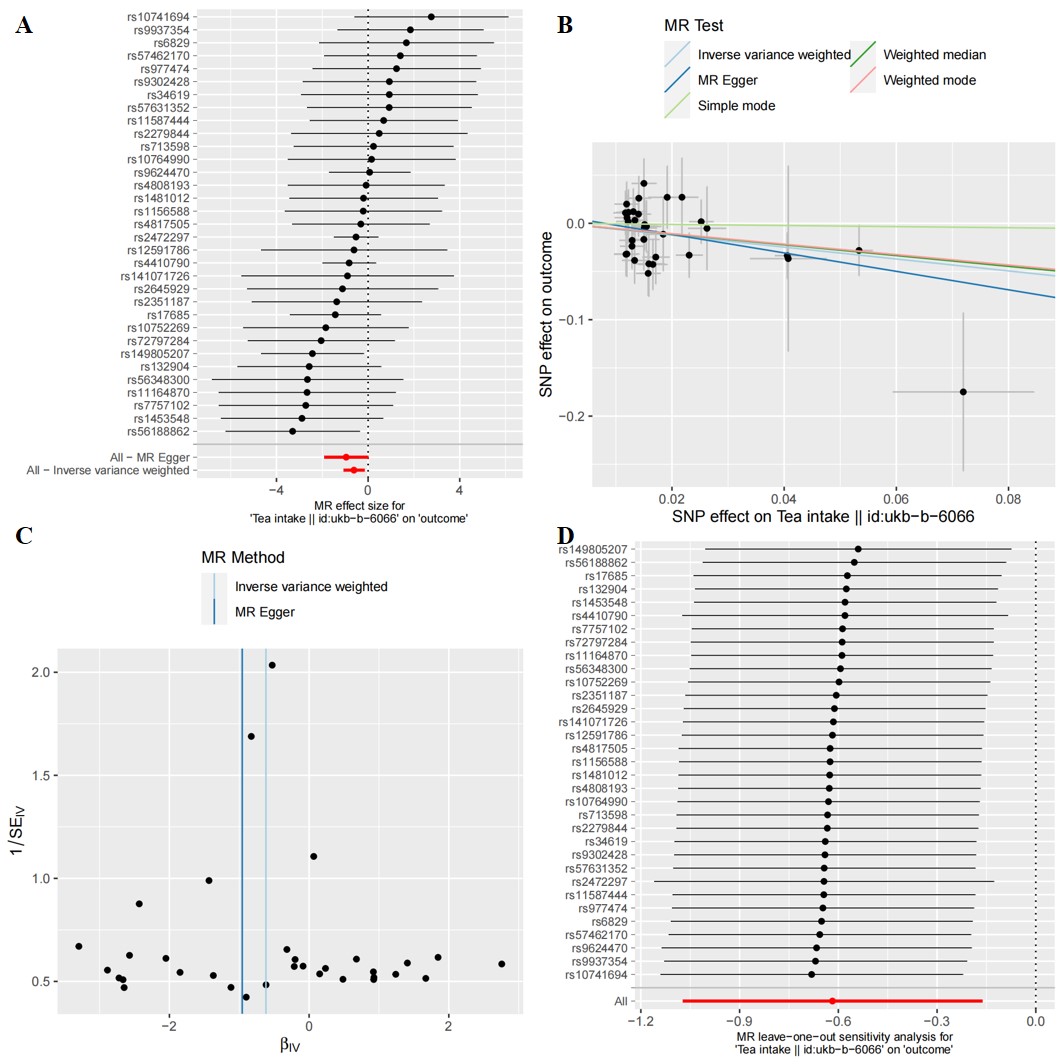
**Figure S5.** Forest plots (A), scatter plots (B), funnel plots (C), and leave-one-out plots (D) of genetically predicted tea intake on cirrhosis.

**Supplementary Figure**


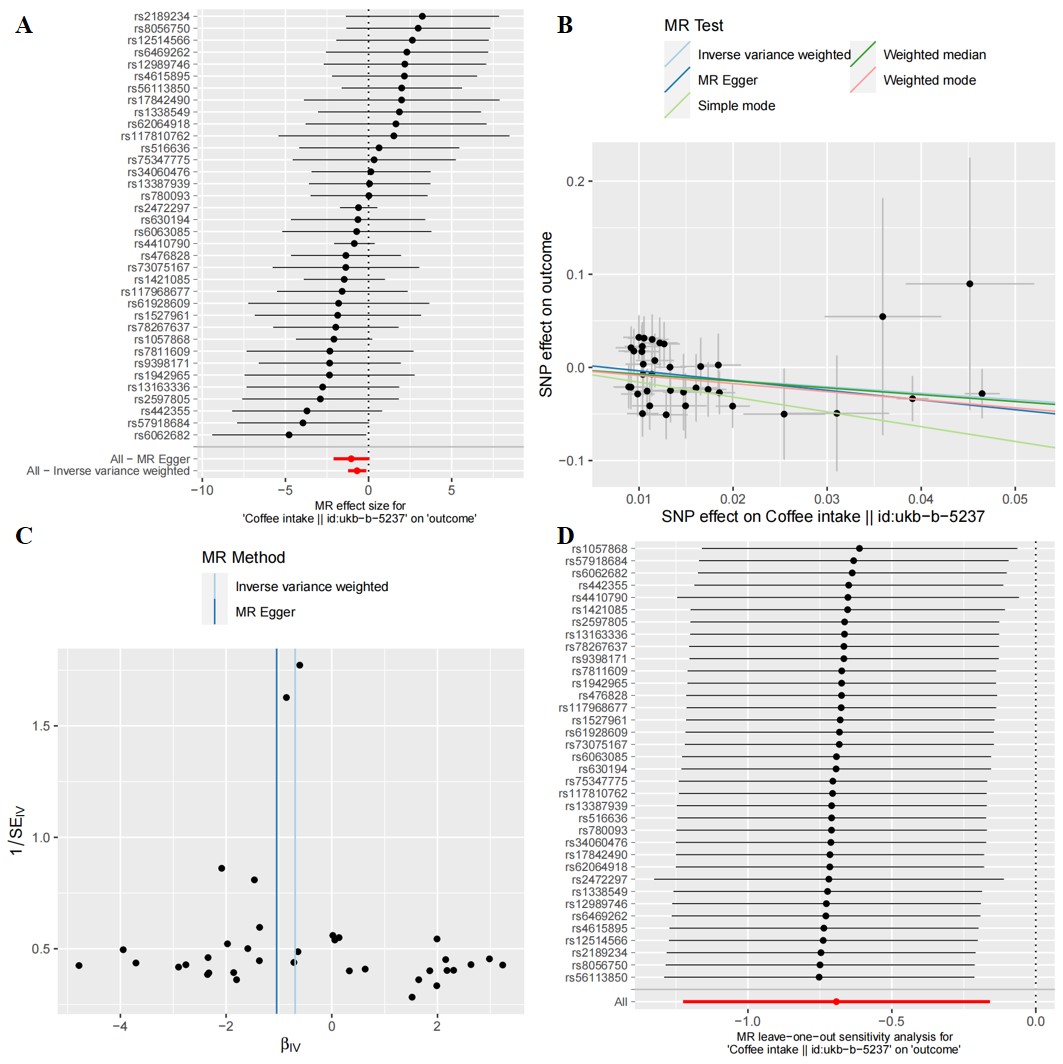
**Figure S6.** Forest plots (A), scatter plots (B), funnel plots (C), and leave-one-out plots (D) of genetically predicted coffee intake on cirrhosis.

**Supplementary Figure**


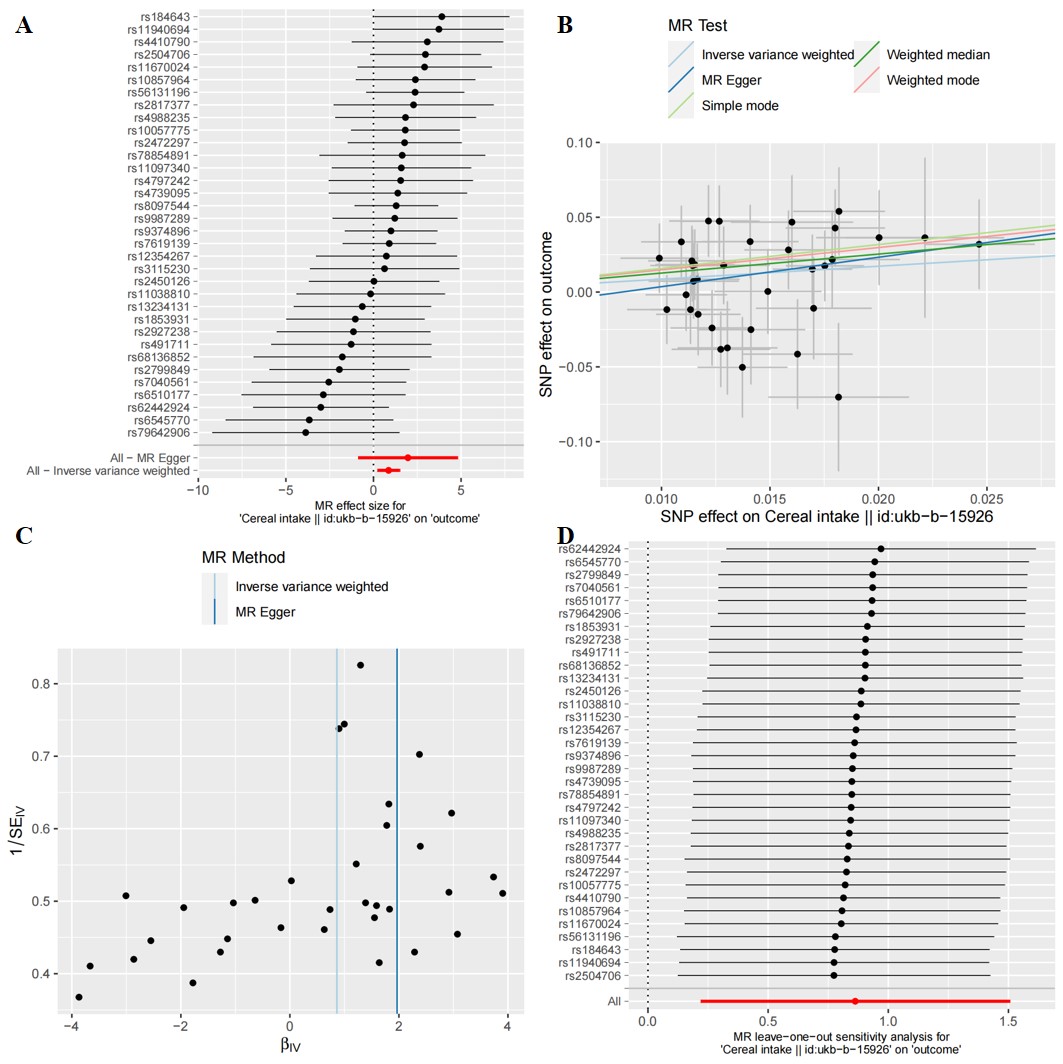
**Figure S7.** Forest plots (A), scatter plots (B), funnel plots (C), and leave-one-out plots (D) of genetically predicted cereal intake on cirrhosis.

**Supplementary Figure**


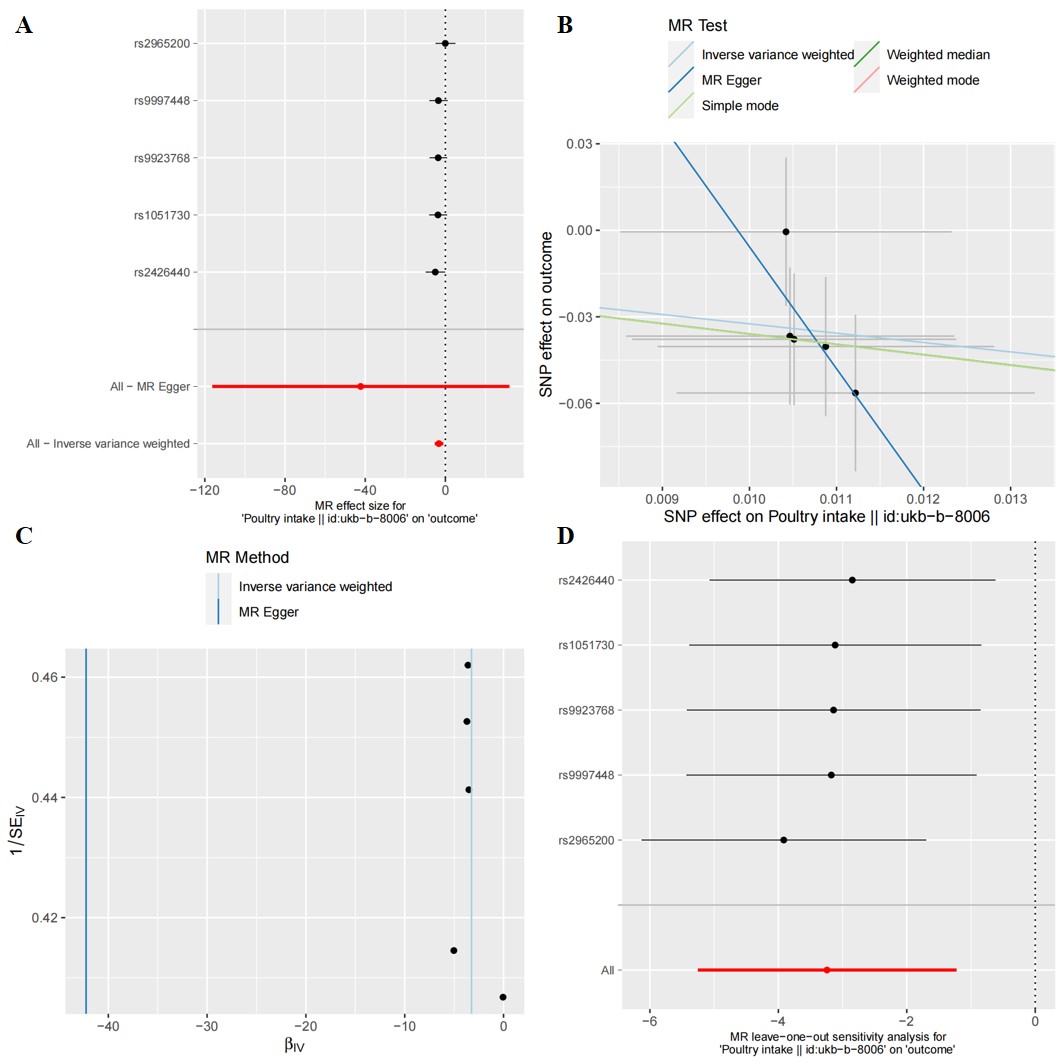
**Figure S8.** Forest plots (A), scatter plots (B), funnel plots (C), and leave-one-out plots (D) of genetically predicted poultry intake on cirrhosis.

**Supplementary Figure**


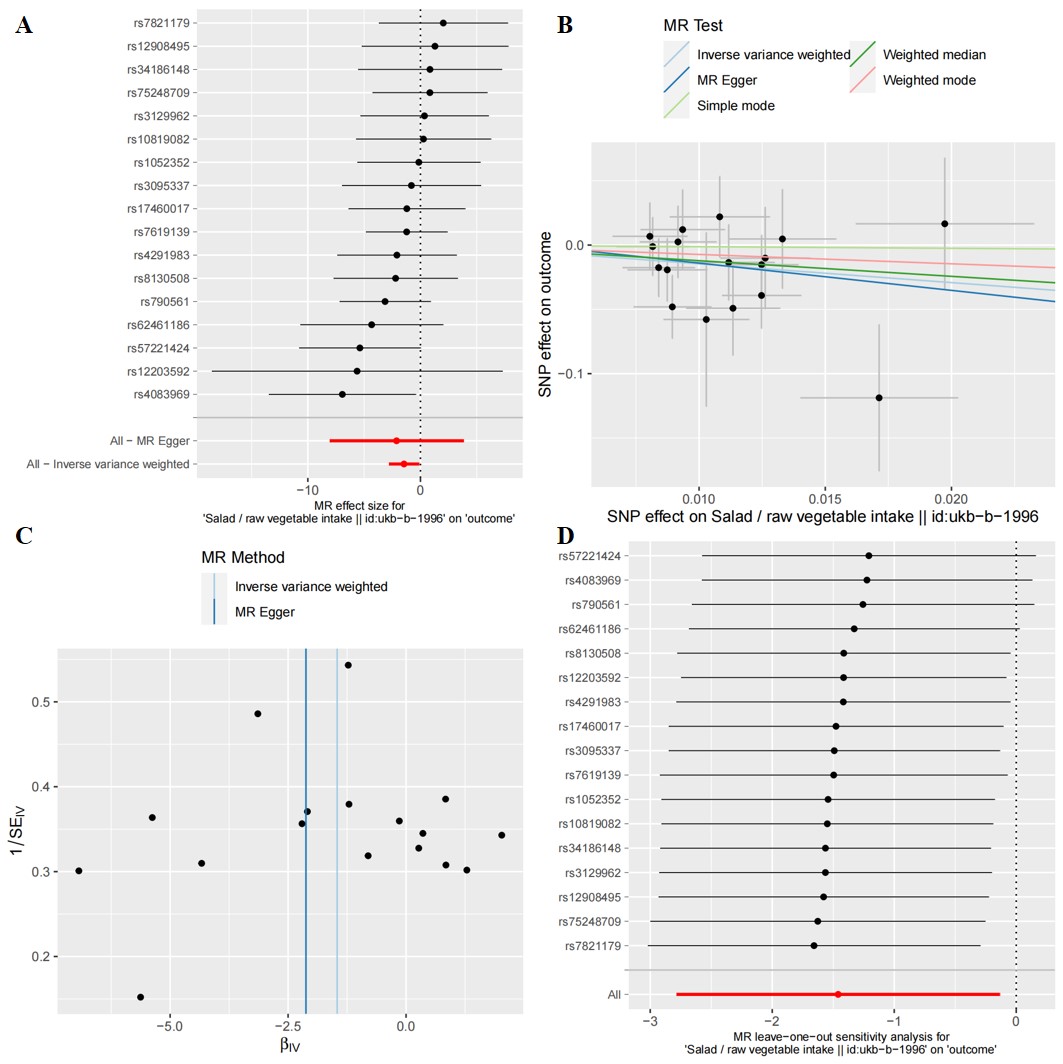
**Figure S9.** Forest plots (A), scatter plots (B), funnel plots (C), and leave-one-out plots (D) of genetically predicted salad / raw vegetable intake on cirrhosis.

**Supplementary Figure**


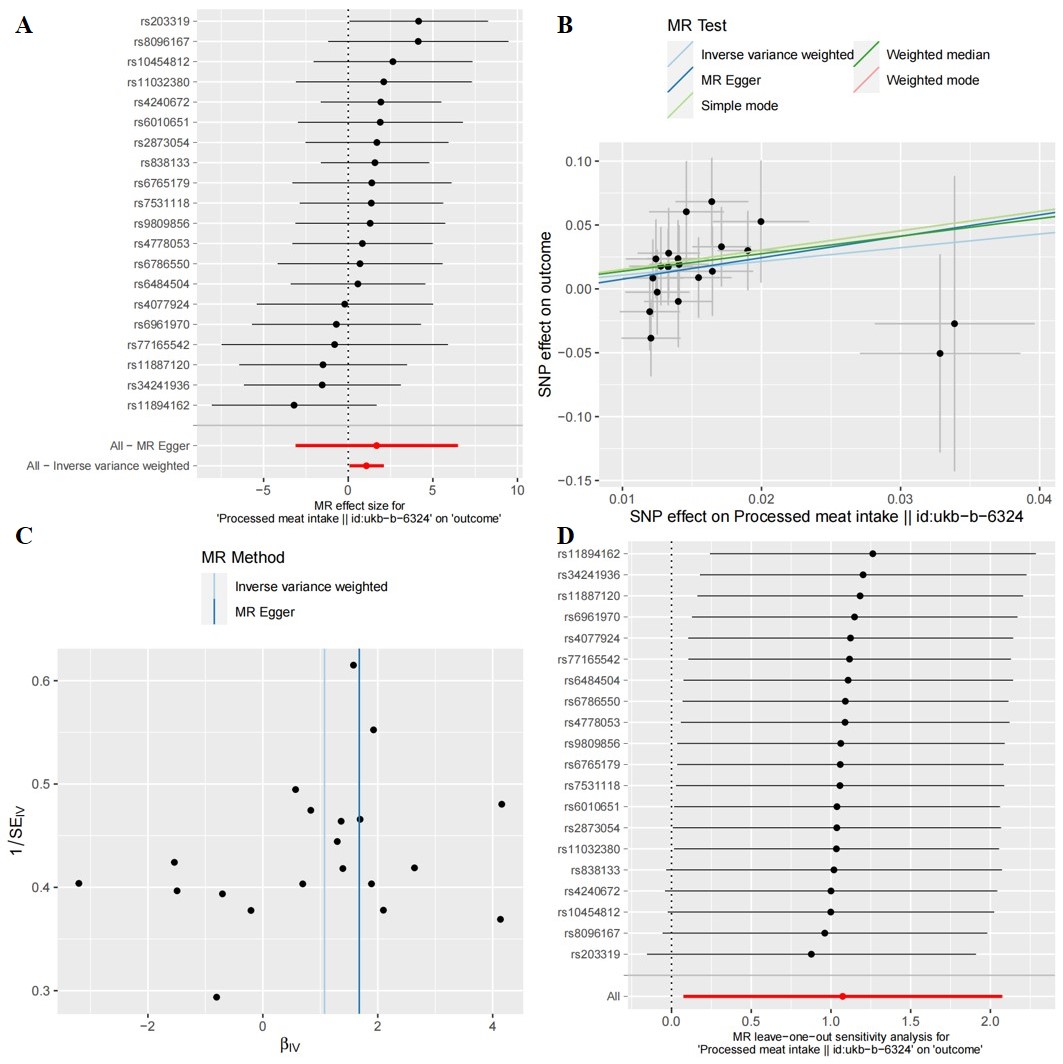
**Figure S10.** Forest plots (A), scatter plots (B), funnel plots (C), and leave-one-out plots (D) of genetically predicted processed meat intake on non-alcoholic fatty liver diseases.

**Supplementary Figure**

**
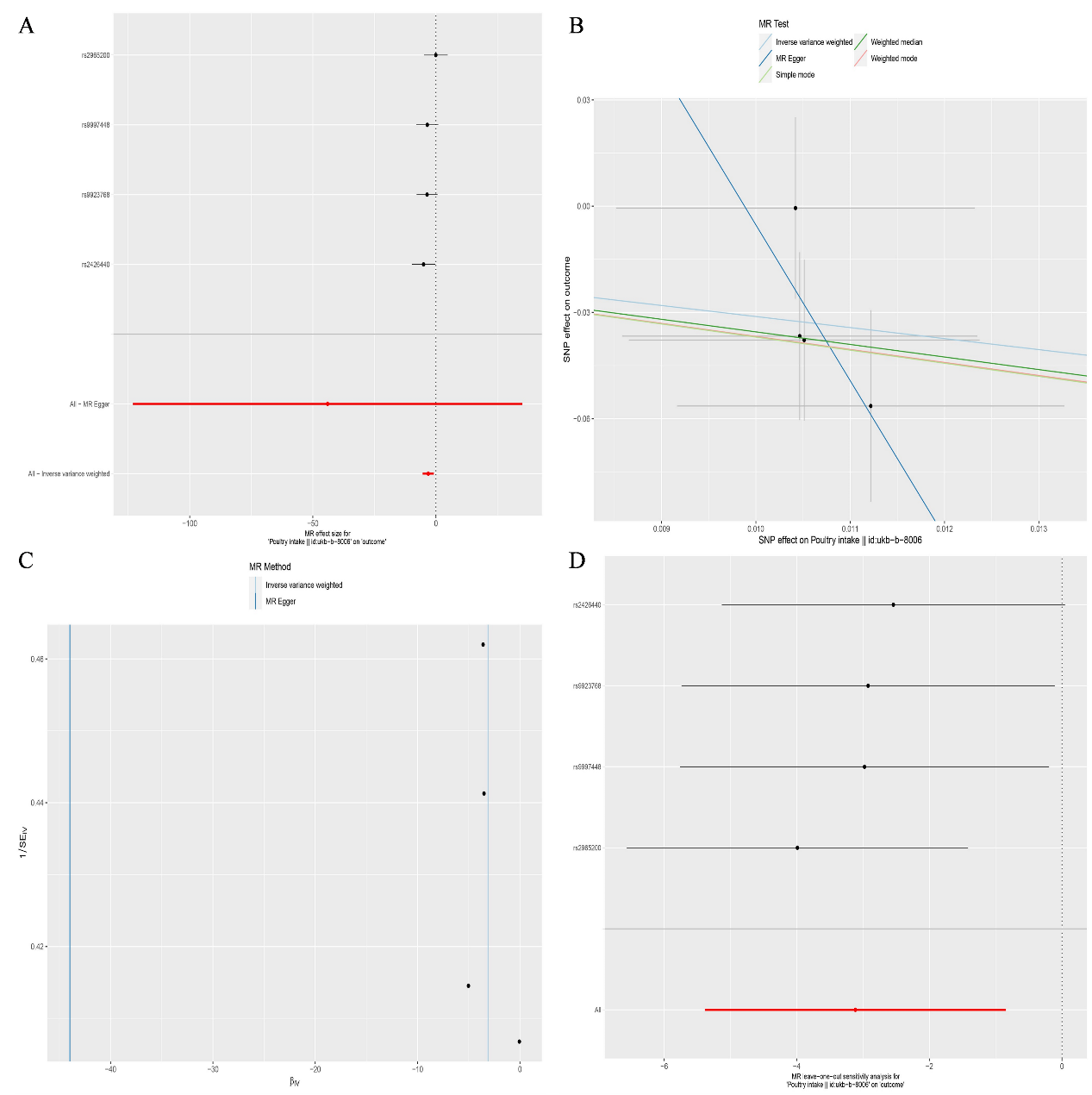
**

**Figure S11.** Forest plots (A), scatter plots (B), funnel plots (C), and leave-one-out plots (D) for adjusted causal association between poultry intake and cirrhosis.

**Supplementary Figure**

**
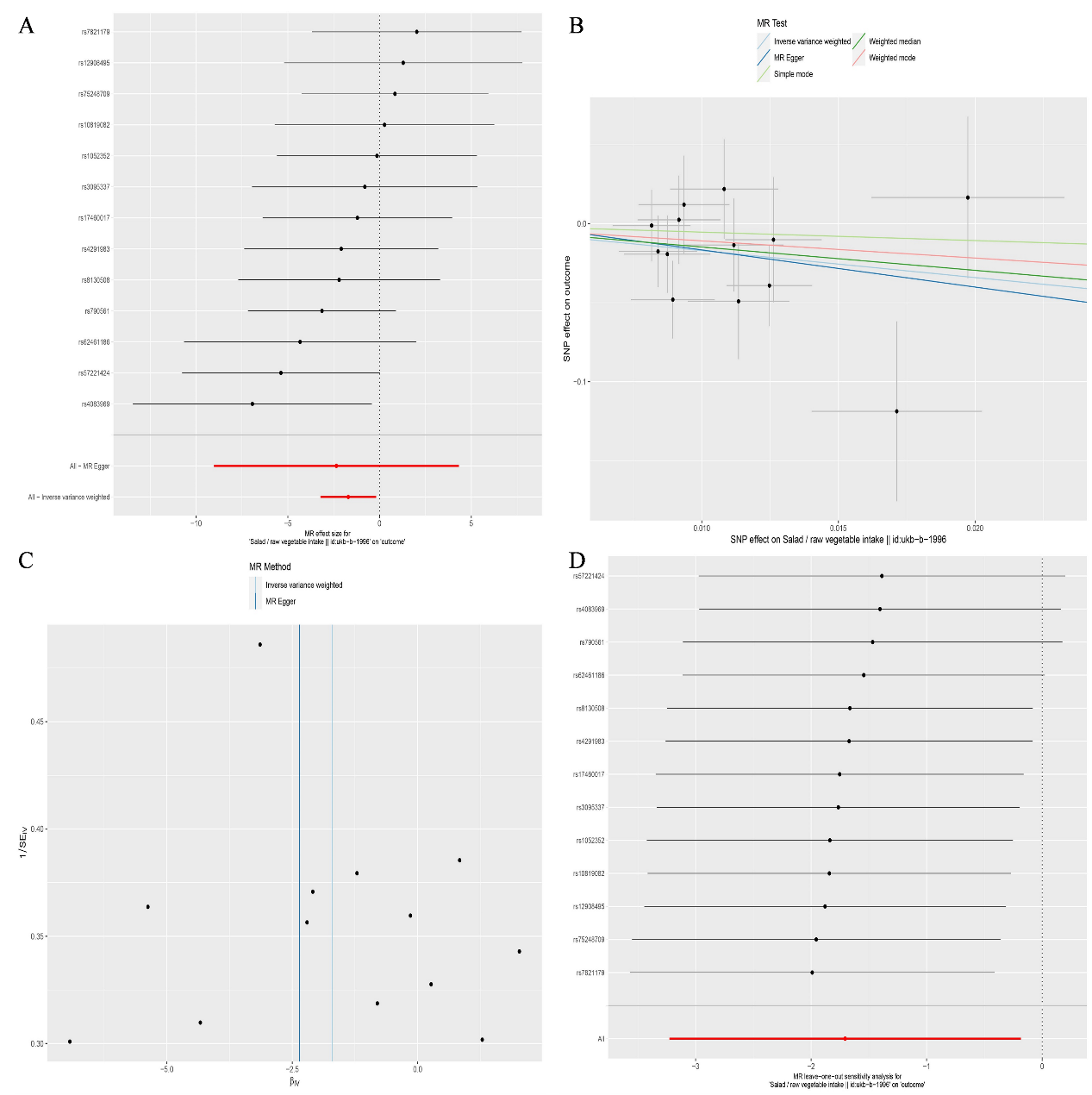
Figure S12**. Forest plots (A), scatter plots (B), funnel plots (C), and leave-one-out plots (D) for adjusted causal association between salad / raw vegetable intake and cirrhosis.
